# Identification of the Carbohydrate and Organic Acid Metabolism Genes Responsible for Brix in Tomato Fruit by Transcriptome and Metabolome Analysis

**DOI:** 10.3389/fgene.2021.714942

**Published:** 2021-09-03

**Authors:** Ning Li, Juan Wang, Baike Wang, Shaoyong Huang, Jiahui Hu, Tao Yang, Patiguli Asmutola, Haiyan Lan, Yu Qinghui

**Affiliations:** ^1^Institute of Horticulture Crops, Xinjiang Academy of Agricultural Sciences, Urumqi, China; ^2^College of Forestry and Horticulture, Xinjiang Agricultural University, Urumqi, China; ^3^Xinjiang Key Laboratory of Biological Resources and Genetic Engineering, College of Life Science and Technology, Xinjiang University, Urumqi, China

**Keywords:** tomato, fruit brix, organic acid, carbohydrate, metabolic, transcriptome, TCA cycle

## Abstract

**Background:**

Sugar and organic acids not only contribute to the formation of soluble solids (Brix) but also are an essential factor affecting the overall flavor intensity. However, the possible metabolic targets and molecular synthesis mechanisms remain to be further clarified.

**Methods:**

UHPLC-HRMS (ultrahigh-performance liquid chromatography and high-resolution mass spectrometry) combined with comparative transcriptome analysis were performed in fruits at green ripe (S1), turning-color (S2), and red ripe (S3) stages of two tomato genotypes TM-1 (*Solanum galapagense* L., LA0436) and TM-38 (*S. lycopersicum* L. cultivar M82, LA3475) that vary in fruit Brix.

**Results:**

The fruit Brix of TM-1 was nearly twice that of TM-38 at S3. Nevertheless, TM-1 accumulated 1.84- and 2.77-fold the L-malic acid and citric acid in red ripe fruit (S3) compared with TM-38, respectively. D-glucose and D-fructose in TM-1 and TM-38 fruits tended to be similar at S3. Concomitantly, the sugar/organic acid ratio of TM-38 fruits were 23. 08-, 4. 38-, and 2.59-fold higher than that of TM-1 fruits at S1, S2, and S3, respectively. Among starch and sucrose (carbohydrate, CHO) metabolism (ko00500) genes, *SUS* (Solyc07g042550.3) and *BAM* (Solyc08g077530.3) were positively (*r* = 0.885–0.931) correlated with the sugar/organic acid ratio. Besides, *INV* (Solyc09g010080.3 and Solyc09g010090.5.1), *AAM* (Solyc04g082090.3), *4-*α*-GTase* (Solyc02g020980.2.1), *BGL2* (Solyc06g073750.4, Solyc06g073760.3, and Solyc01g081170.3), *TPS* (Solyc01g005210.2 and Solyc07g006500.3), and *TPP* (Solyc08g079060.4) were negatively (*r* = −0.823 to −0.918) correlated with the sugar/organic acid ratio. The organic acid (TCA cycle) metabolism (ko00020) gene *ALMT* (Solyc01g096140.3) was also negatively (*r* = −0.905) correlated with the sugar/organic acid ratio.

**Conclusion:**

Citric acid may play a more dominant role in the sugar/organic acid ratio of the tomato fruit, and the contribution of both L-malic acid and citric acid to the fruit Brix was much greater than that of D-glucose and D-fructose. Genes involved in CHO and TCA metabolism, which have a significant correlation with the sugar/organic acid ratio were considered to be the contributing factors of fruit Brix.

## Introduction

Tomato (*Solanum lycopersicum*) is one of the most popular and valuable fruits with limited caloric supply, and an excellent source of fiber, minerals, phenols, vitamins A, C, E, and lycopene, making it an excellent “functional food” meeting basic nutritional requirements (Dorais et al., 2008; Beckles et al., 2011; Giovannetti et al., 2012). Total soluble solids (TSS) is the most important fruit quality parameter in both fresh market and processed tomatoes, indicating the proportion (%) of dissolved solids in a solution (Schaffer et al., 1999; Xu et al., 2018). It is the sum of sugars (sucrose and hexoses; 65%), acids (citrate and malate; 13%), and other minor components (phenols, amino acids, soluble pectins, ascorbic acid, and minerals) in the tomato fruit pulp (Kader, 2008; Beckles, 2012). Sugars and organic acids not only contribute to the improvement of TSS (Brix), one of the key parameters in tomato processing but also play an essential role in overall flavor intensity (Barickman et al., 2016; Carlos et al., 2018). The aim of this study was to compare the contents of organic acids and carbohydrates in two tomato genotypes with drastically different fruit Brix, and to further elucidate a correlation of carbohydrate and organic acids with gene expression levels, which would supply a reference of molecular-assisted selection of high fruit Brix tomato germplasm resources.

Tomato fruit taste and quality vary with varieties, growing conditions, production methods, harvest, and storage time (Langlois et al., 1996; Giovannetti et al., 2012). Natural biodiversity provides opportunities to investigate the multitude of characteristics that affect plant growth and development. Tomato has been an introgression model for valuable traits from wild species (Rick, 1974). Wild plant species offer a way to understand the genetic basis of past domestication events and polymorphisms, providing a basis for breeding superior varieties in the future (Ofner et al., 2016). *S. lycopersicum* is known to hybridize easily with the wild relatives, which can provide valuable sources for the improvement of important agronomic traits (Koblitz, 1991; Schauer et al., 2005). The fruit quality of *S. lycopersicum* is associated with various parameters, including appearance, color intensity, size, shape, flavor, nutritional value, and texture, which ultimately determine acceptability for the consumer (Zhu et al., 2018; Verma et al., 2020). The intensity of tomato flavor is mainly determined by the amount of sugars and organic acids (Çolaka et al., 2020).

The precursor substance ADP-glucose of starch synthesis is catalyzed by the rate-limiting enzyme of ADP-Glc pyrophosphorylase (AGPase), and starch is mainly degraded into reducing sugars under acidic conditions by the action of starch phosphorylase (SP) (Sweetlove et al., 1999). To be metabolized, starch can be hydrolyzed into sucrose to release energy, and sucrose can also form starch to store energy (Gifford et al., 1984; Roby et al., 2002). Sucrose, as a non-reducing sugar, can be decomposed by invertase (INV; EC 3.2.1.26) to form reducing glucose and fructose, and also be reacted with UDP by sucrose synthase (SUS; EC 2.4.1.13) to form uridine diphosphate glucose (UDPG) and fructose (Ruan, 2014; Wan et al., 2018). Sucrose-phosphate synthase (SPS; EC 2.4.1.14) is a key enzyme for catalyzing the conversion of UDP-glucose and fructose-6-phosphate into sucrose-6-phosphate, and sucrose-phosphatase (SPP) can convert sucrose-6-phosphate into sucrose (Grof et al., 1998; Wind et al., 2010; Hashida et al., 2016). UDPG is both a precursor substance of starch synthesis and is also affected by uridine diphosphate glucose pyrophosphorylase (UGPase) forms glucose-1-phosphate to promote the re-synthesis of sucrose (Menendez et al., 2002; Finlay et al., 2003). Through amylase (AMY) activity, starch can also yield maltose, which is exported to the cytoplasm and cleaved to produce glucose monomers (Weise et al., 2004). Although glucose and fructose are interconverted after phosphorylation, glucose is more preferentially used than fructose in several plant cells (Kandel-Kfir et al., 2006). After phosphorylation, glucose and fructose are used for the growth or synthesis of storage materials: sucrose and starch (Krook et al., 1998). On the other aspect, organic acids are key factors in maintaining pH and changing the sensory quality of fruit, and the evaluation of fruit maturity and the quality of a particular variety depends on the sugar/organic acid ratio (Jie et al., 2018). The genes encoding the aluminum-activated malate transporter have often been reported to be involved in the regulation of the organic acid levels (Jawad et al., 2020; Umer et al., 2020).

Since sugars and organic acids function as signaling molecules in many developmental processes throughout the plant life cycle, uncovering these functions and their interactions with other signaling pathways presents a formidable challenge (Lastdrager et al., 2014; Li et al., 2016; Jawad et al., 2020). A new technology such as transcriptome has been used to uncover the genes involved in starch and sucrose (carbohydrate, CHO) and organic acid (TCA) metabolisms (Zhang et al., 2015; Umer et al., 2020). Therefore, a potential mechanism for identifying key candidate genes responsible for divergent fruit Brix content is presented in this study. Metabolite profiles evaluated using UHPLC-HRMS (ultrahigh-performance liquid chromatography and high-resolution mass spectrometry) and transcript level of selected genes coding for enzymes metabolism were determined in fruits of TM-1 (*S. galapagense* L., LA0436) and TM-38 (*S. lycopersicum* L. cultivar M82, LA3475) at three developmental stages.

## Materials and Methods

### Plant Materials

Two tomato genotypes, differing in carbohydrate content, TM-1 (*S. galapagense* L., LA0436), and TM-38 (*S. lycopersicum* L. cultivar M82, LA3475) were introduced from the UC Davis/C.M. Tomato Genetics Resource Center (TGRC) and maintained by the Department of Plant Science, University of California, Davis, CA 95616. The TGRC undertook formal identification of the samples, provided details of the specimens deposited and allowed the collection. Both tomato genotypes were grown in the Anningqu experimental station of the Xinjiang Academy of Agricultural Sciences (87°49′63″N, 43°95′16″E; altitude: 680–920 m). Briefly, six fruits from each of the two strains were randomly sampled as a biological repeat at green ripe stage (S1), turning-color period (S2), and red ripe (S3) stage, respectively. There were three biological replicates for each genotype. All fruit samples were immediately frozen in liquid nitrogen and stored at −80°C for further physical, metabolic, and gene expression evaluation.

### Determination of Fruit Diameter and Total Soluble Solids

The determination of fruit diameter and TSS was based on the tomato fruit at S3. The transverse and longitudinal diameters were measured by a vernier caliper with an accuracy of 0.01 mm (Mitutoyo CD-15CPX, Japan). TSS was measured by refractometer sugar sweetness meter (Guangzhou Weilai Electronic Technology Co., Ltd.).

### Metabolite Extraction for Ultrahigh-Performance Liquid Chromatography and High-Resolution Mass Spectrometry

Lyophilized tomato fruit samples were finely ground, and a certain amount of powdered samples (see Data Sheet 1) were placed into EP tubes. Then an appropriate amount of extraction solution (10% methanol, see Data Sheet 1 for specific volume) was added. After vortex mixing for 30 s, steel balls were added and ground for 4 min at 45 Hz in a Retsch^®^ Mixer Mill MM400 (Retsch, Haan, Germany), then ultrasonicated for three times and incubated in ice water every 5 min. After centrifugation at 13,000 × *g* for 15 min at 4°C, the supernatant was transferred for the UHPLC-HRMS analysis.

### Targeted Metabolomics Profiling of Ultrahigh-Performance Liquid Chromatography and High-Resolution Mass Spectrometry

UHPLC-HRMS analyses were performed using Waters ACQUITY UPLC (Waters, Millford, MA, United States) ultrahigh-performance liquid chromatograph equipped with a Waters ACQUITY UPLC BEH C_18_ column (100 mm × 2.1 mm, 1.7 μm, Waters) to separate the target compounds. Full scan mass spectrometry was performed by XEVO G2XS Q-TOF high-resolution mass spectrometer. The ion source parameters are as follows: capillary voltage = 2,000 V, sampling cone = 40 V, source temperature = 115°C, desolvation temperature = 500°C, and desolvation gas = 900 L/h. For each target compound, the parent ions under high-resolution (QTOF) conditions were used for quantitative analysis. The specific parameters [retention time (RT), mass-to-charge ratio (m/z), and polarity] are shown in Supplementary Table 2. The calibration curve is shown in Data Sheet 2; y is the peak area of the target compound, and x is the concentration of the target compound (μg/ml). The least square method was used for regression analysis. When the weight was set at 1/x, the calibration solution recovery rate (accuracy) and correlation coefficient (*R*^2^) were the best. If the signal-to-noise ratio (S/N) of a calibration concentration is close to 20, or the recovery rate exceeds the range of 80–120%, the calibration point of the concentration was excluded.

### RNA Isolation

Total RNA was isolated from approximately 200 mg of lyophilized tomato fruit samples collected at 45 DAF using an RNAprep Pure Plant Plus Kit (TIANGEN, Beijing, China). Then the quality and quantity of the purified RNA samples were preliminarily characterized by Multiskan Go Full Wavelength Microplate Spectrophotometer (Thermo Fisher Scientific, MA, Waltham, United States).

### RNA-seq and Differential Gene Expression Analysis

The six triplicate samples (TM-1 and TM-38 at three developmental stages) yielded 18 non-directional cDNA libraries with a total of 121.92 Gb of clean data (Table 1) using illumina HiSeq 2500 platform by signal end read libraries method of the SBS (Sequencing By Synthesis) technology, which was performed at the Biomarker Technologies Co., Ltd. (Beijing, China). The raw reads were cleaned, and the clean reads were aligned onto the tomato reference genome^1^. During the detection process of differentially expressed genes (DEGs), fold change > 2 and false discovery rate (FDR) < 0.01 was used as the screening standard. Gene expression was scaled using values of the fragments per kilobase of exon per million mapped reads (FPKM) ≥ 1.0 as a threshold to identify significant DEGs (Kandel-Kfir et al., 2006). The Gene Ontology (GO)^2^ and the Kyoto Encyclopedia of Genes and Genomes (KEGG)^3^ databases were used to assign tomato genes for GO categories and KEGG pathway analyses, respectively.

**TABLE 1:** Summary of RNA-Seq data and mapping metrics.

**Variety**	**Replicate**	**Total reads**	**Clean reads**	**Mapped reads**	**% ≥ Q30**
TM-1-S1	1	46,037,248	23,018,624	43,430,222 (94.34%)	93.21%
	2	39,150,626	19,575,313	36,351,508 (92.85%)	93.88%
	3	52,161,828	26,080,914	48,816,501 (93.59%)	93.68%
TM-1-S2	1	42,747,216	21,373,608	37,308,962 (87.28%)	93.67%
	2	46,262,136	23,131,068	40,358,796 (87.24%)	93.94%
	3	45,726,338	22,863,169	38,948,767 (85.18%)	93.21%
TM-1-S3	1	42,891,178	21,445,589	40,301,779 (93.96%)	93.78%
	2	48,810,696	24,405,348	45,831,719 (93.90%)	93.72%
	3	46,020,758	23,010,379	43,113,028 (93.68%)	92.13%
TM-38-S1	1	43,410,496	21,705,248	40,664,031 (93.67%)	93.65%
	2	45,692,424	22,846,212	42,741,586 (93.54%)	93.71%
	3	44,372,464	22,186,232	41,571,244 (93.69%)	93.64%
TM-38-S2	1	42,858,120	21,429,060	39,913,732 (93.13%)	93.86%
	2	45,430,462	22,715,231	42,228,260 (92.95%)	93.71%
	3	49,700,956	24,850,478	46,521,555 (93.60%)	93.99%
TM-38-S3	1	49,868,822	24,934,411	46,489,230 (93.22%)	93.79%
	2	44,414,518	22,207,259	41,241,871 (92.86%)	93.44%
	3	41,359,802	20,679,901	38,359,691 (92.75%)	93.94%

### RNA-seq Results Verification by Using Quantitative Reverse-Transcription PCR

The cDNA synthesis was conducted with total RNA using RNeasy Mini Kit (QIAGEN, GmbH, Hilden, Germany). Primers (Supplementary Table 2) were designed and synthesized by Sangon Biotech (Shanghai) Co., Ltd. (Shanghai, China). qRT-PCR assays were performed with Quanti Nova SYBR Green PCR Kit (QIAGEN) according to the instructions. Three biological and three technical replicates for each reaction were analyzed on a LightCycler^®^ 96 SW 1.1 instrument (Roche). All relative expression levels of individual genes were normalized by comparing with TM-38 expression at S1 and calculated using the 2^–ΔΔC^T method (Livak and Schmittgen, 2001).

### Statistical Analysis

The Pearson correlation coefficient (*r*) was calculated for correlation analysis, and a two-tailed test was carried out. Origin 9.0 software was used to draw line charts and histograms; Heml software was used to generate heat maps.

## Results

### Characterization of the Ripening Parameters in TM-1 and TM-38

**FIGURE 1:**
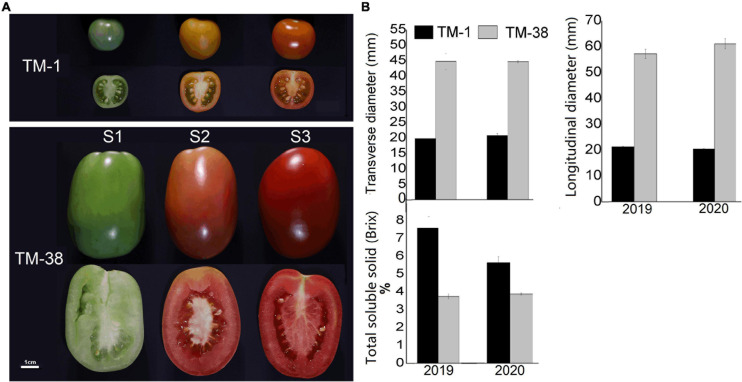
**(A)** Close-up views of TM-1 (*Solanum galapagense* L., LA0436) and TM-38 (*S. lycopersicum* L. cultivar M82, LA3475) at green ripe (S1), turning-color (S2), and red ripe (S3) stages. **(B)** The changes in fruit diameters and total soluble solids (TSS) at S3. Values are means ± SE.

TM-1 is a round fruit with thick skin and orange peel, TM-38 is an oval fruit with red peel and pink flesh (Figure 1A). The transverse and longitudinal diameters of the TM-38 fruits at S3 were 2.2- to 3.0-fold higher than that of the TM-1 fruits in 2019 and 2020 (Figure 1B). In contrast, the TSS of the TM-1 fruits at S3 were 7.6 and 5.7% Brix in 2019 and 2020, which were 2.0- and 1.5-fold higher than that of the TM-38 fruits, respectively.

### Target Metabolite Profiles During Fruit Ripening and Development in TM-1 and TM-38

**FIGURE 2:**
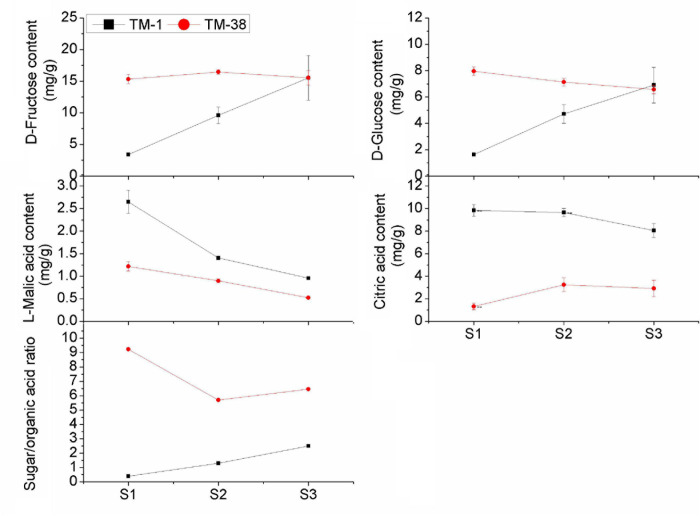
Developmental profiles of D-fructose, D-glucose, L-malic acid, citric acid contents, and the sugar/organic acid ratio in TM-1 and TM-38 at three developmental stages. Values are means ± SE.

The overall trend of D-fructose and D-glucose contents in the TM-1 fruits increased from S1 to S3 (Figure 2). Nevertheless, L-malic acid and citric acid contents displayed an opposite trend. The contents of D-glucose and L-malic acid in the TM-38 fruits decreased from S1 to S3, whereas the contents of D-fructose and citric acid increased first and then decreased from S1 to S3. D-fructose in the TM-38 fruits was 4.55- and 1.72-fold higher than that in the TM-1 fruits at S1 and S2, respectively. Similarly, D-glucose in the TM-38 fruits was 4.89- and 1.51-fold higher than that in the TM-1 fruits at S1 and S2, respectively. However, the concentrations of D-fructose and D-fructose in the TM-1 and TM-38 fruits at S3 were similar. In contrast, the content of L-malic acid in the TM-1 fruits was 2. 17-, 1. 57-, and 1.84-fold higher than that in the TM-38 fruits at the three evaluated developmental stages, respectively. The content of citric acid in the TM-1 fruits was 7. 52-, 2. 97-, and 2.77-fold higher than that in the TM-38 fruits at the three developmental stages, respectively. Besides, the sugar/organic acid ratio of the TM-1 fruits increased from S1 to S3, whereas in the TM-38 fruits, it decreased. However, the sugar/organic acid ratio of the TM-38 fruits were 23. 08-, 4. 38-, and 2.59-fold higher than that of the TM-1 fruits at S1, S2, and S3, respectively.

### Transcriptome Profiling of the TM-1 and TM-38 Fruits

Triplicate sampling of the TM-1 and TM-38 fruits at the three developmental stages yielded 18 RNA samples for transcriptome analysis; a total of 121.92 Gb of clean data were obtained (Table 1). The clean data of each sample reached 5.84 Gb, and the percentage of Q30 base was more than 92.13%. The clean reads of each sample were aligned with the tomato reference genome(see text footnote 1), and the mapping rate ranged from 85.18 to 94.34%.

### Digital Analysis of Differentially Expressed Genes Between the TM-1 and TM-38 Fruits at the Three Developmental Stages

**FIGURE 3:**
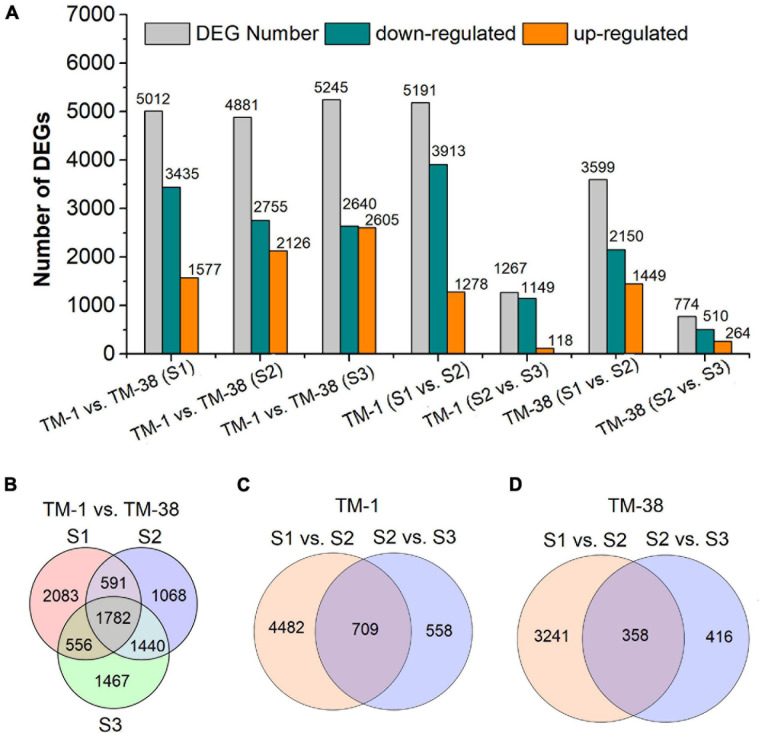
Summary of the number of differentially expressed genes (DEGs) identified by RNA-seq analysis in tomato fruits of TM-1 and TM-38 at three developmental stages. The number of total DEGs, upregulated DEGs, and downregulated DEGs are presented by histograms **(A)**. The Venn diagrams represents the number of common DEGs shared by TM-1 vs. TM-38 at three developmental stages **(B)**, S1 vs. S2 and S2 vs. S3 of TM-1 **(C)**, and TM-38 **(D)**.

Seven pairwise transcriptome comparisons [i.e., TM-1 vs. TM-38 at S1, S2 and S3, TM-1 (S1 vs. S2), TM-1 (S2 vs. S3), TM-38 (S1 vs. S2), TM-38 (S2 vs. S3)] were performed to identify DEGs in the TM-1 and TM-38 fruits at the three developmental stages (Figure 3 and Table 2). The number of DEGs in TM-1 vs. TM-38 was very large in the three developmental stages, accounting for 5,012, 4,881, and 5,245 transcripts, respectively (Figure 3A). There were also more DEGs in S1 vs. S2 of TM-1 and TM-38, which were 5,191 and 3,599, respectively. However, S2 vs. S3 of TM-1 and TM-38 had less DEGs (1,267 and 774, respectively). Among them, the number of DEGs shared by TM-1 vs. TM-38 at S1, S2, and S3 were 1,782 (Figure 3B), while that of S1 vs. S2 and S2 vs. S3 of TM-1 and TM-38 were 709 and 358, respectively (Figures 3C,D). On the whole, there were more downregulated genes than upregulated genes. The number of DEGs annotated to COG, GO, KEGG, KOG, NR, Pfam, Swiss-Prot, and eggNOG databases are also presented in Supplementary Table 3.

**TABLE 2:** Correlation analysis of mean FPKM value of the starch and sucrose metabolism (ko00500) genes with D-fructose, D-glucose, L-malic acid, and citric acid contents, respectively.

**Gene name**	**Accession No.**	**KEGG annotation**	**D-fructose content**	**D-glucose content**	**L-malic acid content**	**Citric acid content**	**Sugar/organic acid ratio**
*SUS*	Solyc03g098290.4	sucrose synthase 5-like [EC 2.4.1.13]	0.519	0.454	–0.402	–0.603	0.484
	Solyc07g042550.3	sucrose synthase [EC 2.4.1.13]	0.596	0.595	–0.594	−0.908*	0.885*
*SPS*	Solyc08g042000.3	sucrose-phosphate synthase A2 [EC 2.4.1.14]	0.416	0.400	–0.483	–0.747	0.728
	Solyc09g092130.3	sucrose-phosphate synthase B [EC 2.4.1.14]	–0.763	–0.652	0.867*	0.618	–0.561
*GAA*	Solyc02g069670.4	alpha-glucosidase-like [EC 3.2.1.20]	–0.090	0.113	0.318	–0.358	0.479
	Solyc04g009630.3	alpha-glucosidase [EC 3.2.1.20]	0.424	0.379	–0.418	0.260	–0.243
*INV*	Solyc03g083910.5	acid beta-fructofuranosidase precursor [EC 3.2.1.26]	–0.116	–0.155	–0.020	0.726	–0.674
	Solyc09g010080.3	beta-fructofuranosidase [EC 3.2.1.26]	–0.805	–0.789	0.686	0.940**	−0.907*
	Solyc09g010090.5.1	cell-wall invertase [EC 3.2.1.26]	−0.866*	−0.827*	0.770	0.940**	−0.881*
	Solyc10g083290.4	acid invertase [EC 3.2.1.26]	−0.970**	−0.939**	0.973**	0.617	–0.617
*BGL1*	Solyc03g119080.4	beta-mannosidase precursor [EC 3.2.1.21]	0.290	0.499	–0.076	–0.389	0.533
	Solyc10g045240.2	vicianin hydrolase [EC 3.2.1.21]	−0.946**	−0.945**	0.946**	0.618	–0.640
	Solyc12g040640.2	beta-glucosidase 44-like isoform X1 [EC 3.2.1.21]	0.142	0.343	0.083	–0.525	0.649
*SP*	Solyc02g077680.4	glycogen phosphorylase 1-like isoform X1 [EC 2.4.1.1]	0.159	0.203	0.039	–0.722	0.671
	Solyc03g065340.3	alpha-1,4 glucan phosphorylase L-1 isozyme [EC 2.4.1.1]	0.147	0.345	0.083	–0.539	0.659
	Solyc05g012510.3	alpha-1,4 glucan phosphorylase L-2 isozyme [EC 2.4.1.1]	–0.037	0.155	0.276	–0.435	0.542
*SSs*	Solyc03g083090.4	soluble starch synthase 1 [EC 2.4.1.21]	0.655	0.560	–0.606	–0.445	0.336
*AAM*	Solyc04g078930.4	alpha-amylase [EC 3.2.1.1]	−0.873*	−0.886*	0.914*	0.573	–0.604
	Solyc04g082090.3	probable alpha-amylase 2 [EC 3.2.1.1]	–0.631	–0.626	0.577	0.958**	−0.918**
*BAM*	Solyc01g094580.3	beta-amylase 7 isoform X2 [EC 3.2.1.2]	–0.589	–0.510	0.617	0.784	–0.672
	Solyc08g077530.3	beta-amylase 3, chloroplastic [EC 3.2.1.2]	0.780	0.847*	–0.566	−0.922**	0.931**
	Solyc09g091030.3	beta-amylase [EC 3.2.1.2]	0.550	0.450	–0.513	–0.737	0.600
*4-*α*-GTase*	Solyc02g020980.2.1	4-alpha-glucanotransferase DPE2-like [EC 2.4.1.25]	−0.973**	−0.963**	0.915*	0.866*	−0.853*
	Solyc04g053120.3	4-alpha-glucanotransferase, chloroplastic/amyloplastic [EC 2.4.1.25]	0.026	0.227	0.201	–0.465	0.583
*AGP*	Solyc01g109790.3	ADP-glucose pyrophosphorylase large subunit [EC 2.7.7.27]	–0.025	0.172	0.256	–0.428	0.544
	Solyc07g019440.3	ADP-glucose pyrophosphorylase large subunit [EC 2.7.7.27]	0.659	0.617	–0.524	−0.901*	0.809
*BGL2*	Solyc01g010390.3	beta-glucosidase 40 [EC 2.4.1.15]	−0.848*	−0.850*	0.875*	0.406	–0.439
	Solyc01g081170.3	unnamed protein product, partial [EC 2.4.1.15]	–0.659	–0.656	0.580	0.971**	−0.916*
	Solyc02g080290.3	beta-glucosidase 18-like [EC 2.4.1.15]	0.205	0.331	–0.136	–0.668	0.749
	Solyc04g015560.4	uncharacterized protein LOC101247513 [EC 2.4.1.15]	–0.639	–0.506	0.780	0.540	–0.424
	Solyc06g005970.2	uncharacterized protein LOC101263519 [EC 2.4.1.15]	0.561	0.508	–0.457	–0.789	0.678
	Solyc06g073740.3	beta-glucosidase BoGH3B-like [EC 2.4.1.15]	0.091	0.078	–0.155	0.569	–0.508
	Solyc06g073750.4	uncharacterized protein LOC101266643 [EC 2.4.1.15]	–0.771	–0.748	0.695	0.971**	−0.911*
	Solyc06g076780.3	uncharacterized protein LOC101260057 [EC 2.4.1.15]	–0.178	–0.007	0.413	–0.381	0.460
	Solyc07g063390.3	beta-glucosidase 18-like isoform X2 [EC 2.4.1.15]	–0.351	–0.378	0.335	0.844*	–0.805
	Solyc07g063880.4	putative beta-glucosidase 41 [EC 2.4.1.15]	−0.983**	−0.961**	0.968**	0.690	–0.690
	Solyc09g075060.3	beta-glucosidase 11-like [EC 2.4.1.15]	0.574	0.524	–0.603	−0.876*	0.799
	Solyc11g008720.3	beta-glucosidase 42 [EC 2.4.1.15]	0.022	–0.065	–0.045	0.535	–0.581
	Solyc11g071640.3	uncharacterized protein LOC101256554 isoform X2 [EC 2.4.1.15]	−0.948**	−0.904*	0.936**	0.838*	–0.796
*TPS/TPP*	Solyc01g005210.2	alpha, alpha-trehalose-phosphate synthase [EC 3.1.3.12]	–0.299	–0.371	0.230	0.846*	−0.846*
	Solyc02g071590.3	alpha, alpha-trehalose-phosphate synthase [EC 3.1.3.12]	−0.943**	−0.945**	0.947**	0.695	–0.709
	Solyc02g072150.3	probable alpha, alpha-trehalose-phosphate synthase [EC 3.1.3.12]	0.485	0.472	–0.567	–0.777	0.761
	Solyc03g007290.4	probable trehalose-phosphate phosphatase 2 [EC 3.1.3.12]	–0.006	0.123	0.162	–0.614	0.662
	Solyc03g083960.3	trehalose-phosphate phosphatase A [EC 3.1.3.12]	0.260	0.441	–0.048	–0.669	0.772
	Solyc04g054930.3	probable trehalose-phosphate phosphatase J [EC 3.1.3.12]	−0.819*	–0.768	0.914*	0.331	–0.331
	Solyc04g072920.4	probable trehalose-phosphate phosphatase J [EC 3.1.3.12]	0.333	0.510	–0.132	–0.708	0.792
	Solyc06g060600.3	probable trehalose-phosphate phosphatase F [EC 3.1.3.12]	−0.918**	−0.922**	0.937**	0.603	–0.627
	Solyc07g006500.3	trehalose-6-phosphate synthase [EC 3.1.3.12]	−0.895*	−0.946**	0.858*	0.783	−0.823*
	Solyc07g062140.3	trehalose-phosphate synthase 1 [EC 3.1.3.12]	0.005	0.204	0.204	–0.476	0.591
	Solyc08g079060.4	probable trehalose-phosphate phosphatase F [EC 3.1.3.12]	−0.986**	−0.971**	0.919**	0.838*	−0.828*
*HXK*	Solyc02g091830.3	probable hexokinase-like 2 protein [EC 2.7.1.1]	–0.243	–0.081	0.493	–0.315	0.382
	Solyc04g081400.3	plastidic hexokinase [EC 2.7.1.1]	–0.315	–0.134	0.597	–0.031	0.134
	Solyc11g065220.2	hexokinase-3-like [EC 2.7.1.1]	–0.090	0.100	0.302	–0.430	0.533
	Solyc12g008510.2	Hexokinase [EC 2.7.1.1]	–0.553	–0.471	0.365	0.605	–0.520
*ALMT*	Solyc01g096140.3	Aluminum-activated malate transporter	−0.870*	−0.848*	0.752	0.943**	−0.905*
	Solyc11g068970.2		–0.537	–0.510	0.409	0.0864*	–0.807
*ICDH*	Solyc01g005560.3	isocitrate dehydrogenase [EC 2.3.3.8]	–0.058	–0.095	–0.057	0.681	–0.649
*CS*	Solyc07g055840.3	citrate synthase [EC 2.3.3.1]	–0.464	–0.431	0.304	0.828*	–0.751
*ACS*	Solyc12g099260.2	ATP-citrate synthase alpha chain protein [EC 1.1.1.42]	−0.832*	–0.772	0.937**	0.382	–0.370
*PDHB-1*	Solyc05g024160.3	pyruvate dehydrogenase E1 component subunit beta-1 [EC 1.2.4.1]	–0.783	–0.698	0.666	0.776	–0.697
*SDH*	Solyc04g055020.2 Solyc04g055030.2	succinate dehydrogenase [ubiquinone] iron-sulfur subunit 3 [EC 1.3.5.1]	–0.673	–0.632	0.503	0.845*	–0.782
			–0.788	–0.748	0.629	0.858*	–0.806

*Significantly correlated at the 0.05 level (bilateral). **Significantly correlated at the 0.01 level (bilateral).

### Identification of Key Processes Responsible for Organic Acid and Carbohydrate Accumulation in the TM-1 and TM-38 Fruits

**FIGURE 4:**
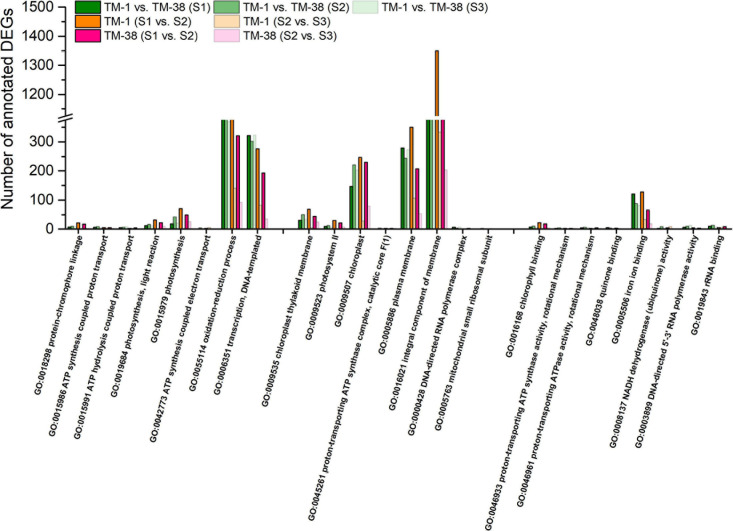
Fisher’s exact test for the significant (top eight) enrichment analysis of Gene Ontology (GO) terms of the biological process, cellular components, and molecular function categories of the annotated DEGs in TM-1 and TM-38 at three developmental stages.

To understand the main functional categories represented by DEGs, GO functional enrichment analysis was performed with all reference genes as the background. The top eight significantly enriched GO terms in TM-1 vs. TM-38 at the three developmental stages are displayed in three main categories: biological process, cellular component, and molecular function (Figure 4). GO terms of photosynthetic electron transport in photosystem II (GO:0009772), protein–chromophore linkage (GO:0018298), ATP synthesis-coupled proton transport (GO:0015986), ATP hydrolysis-coupled proton transport (GO:0015991), response to herbicide (GO:0009635), photosynthesis, light reaction (GO:0019684), photosynthesis (GO:0015979), ATP synthesis-coupled electron transport (GO:0042773) and transcription, and DNA templated (GO:0006351) in the biological process category were shared in TM-1 vs. TM-38 at the three developmental stages. In the cellular component category, GO terms of chloroplast thylakoid membrane (GO:0009535), photosystem II (GO:0009523), chloroplast (GO:0009507), proton-transporting ATP synthase complex, catalytic core F(1) (GO:0045261), plasma membrane (GO:0005886), photosystem II reaction center (GO:0009539), and mitochondrial small ribosomal subunit (GO:0005763) were observed in TM-1 vs. TM-38 at the three developmental stages. In the molecular function category, electron transporter, transferring electrons within the cyclic electron transport pathway of photosynthesis activity (GO:0045156), chlorophyll-binding (GO:0016168), proton-transporting ATP synthase activity, rotational mechanism (GO:0046933), proton-transporting ATPase activity, rotational mechanism (GO:0046961), oxygen evolving activity (GO:0010242), quinone binding (GO:0048038), iron ion binding (GO:0005506), NADH dehydrogenase (ubiquinone) activity (GO:0008137), DNA-directed 5′–3′ RNA polymerase activity (GO:0003899), and rRNA binding (GO:0019843) were also shared in TM-1 vs. TM-38 at the three developmental stages.

**FIGURE 5:**
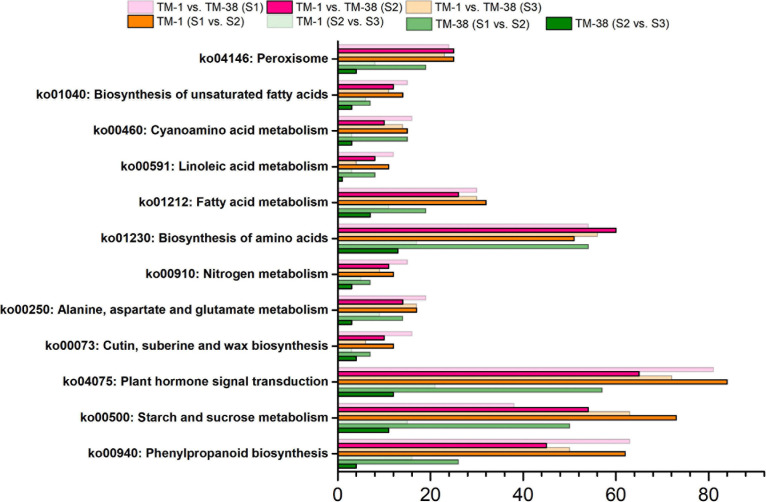
Kyoto Encyclopedia of Genes and Genomes (KEGG) enrichment analysis of the annotated DEGs in TM-1 and TM-38 at three developmental stages.

Kyoto Encyclopedia of Genes and Genomes analysis was performed to further systematically understand the molecular interactions among the DEGs, the top 12 KEGG pathways with a *p*-value ≤ 0.01 were found to be significantly enriched (Figure 5). The significantly enriched KEGG pathways of CHO metabolism (ko00500) were shared in TM-1 vs. TM-38 at the three developmental stages. In addition, although the TCA cycle metabolism (ko00020) was not significantly enriched, it has been reported to be closely related to organic acid metabolism (Umer et al., 2020). Therefore, the CHO and TCA metabolisms were selected for subsequent analysis.

### Transcriptional Expression Analysis of Differentially Expressed Genes Involved in Carbohydrate and TCA Cycle Metabolisms

**FIGURE 6:**
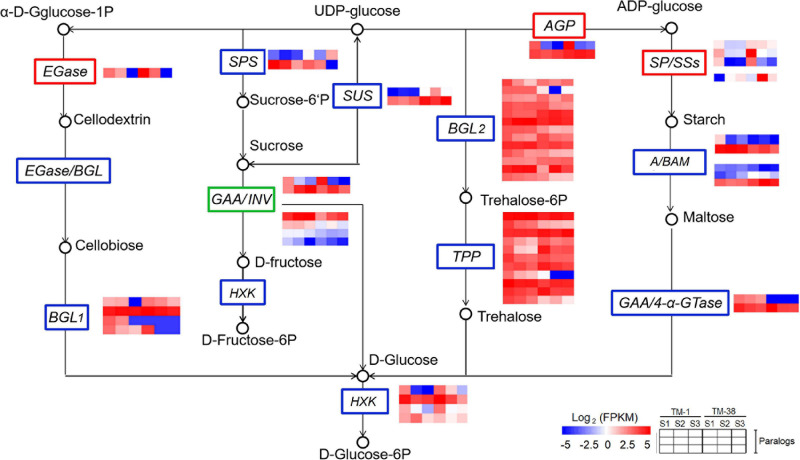
Differential expression of genes involved in starch and sucrose metabolism pathway in TM-1 and TM-38 at three developmental stages. Heat maps depict the normalized gene expression values, which represent the means ± SD of three biological replicates. Expression values of 18 libraries are presented as FPKM normalized log_2_-transformed counts.

**FIGURE 7:**
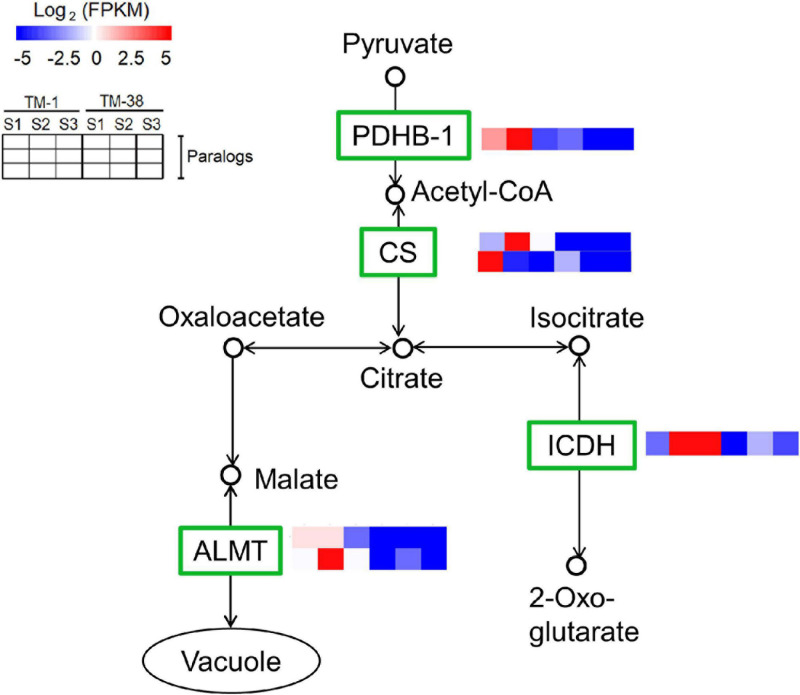
Differential expression of genes involved in citrate cycle (TCA cycle) pathway in TM-1 and TM-38 at three developmental stages. Heat maps depict the normalized gene expression values, which represent the means ± SD of three biological replicates. Expression values of 18 libraries are presented as FPKM normalized log_2_-transformed counts.

The transcriptional expression of CHO metabolism genes in the TM-1 and TM-38 fruits at the three developmental stages was investigated by preparing heat maps (Figure 6). Two *SUSs* (Solyc03g098290.4 and Solyc07g042550.3), one *SPS* (Solyc08g042000.3), three *SPs* (Solyc02g077680.4, Solyc03g065340.3, and Solyc05g012510.3), one soluble starch synthase (*SSs*; Solyc03g083090.4), two beta-amylase (*BAMs*; Solyc08g077530.3 and Solyc09g091030.3), two ADP-glucose pyrophosphorylase large subunit (*AGPs*; Solyc01g109790.3 and Solyc07g019440.3), three beta-glucosidase (*BGL2s*; Solyc06g005970.2, Solyc06g076780.3, and Solyc09g075060.3), one trehalose-phosphate synthase (*TPS*; Solyc02g072150.3), three trehalose-phosphate phosphatases (*TPPs;* Solyc03g007290.4, Solyc03g083960.3, and Solyc04g072920.4), and two hexokinases [*HXKs*; (Solyc02g091830.3 and Solyc11g065220.2] were significantly upregulated in the TM-38 fruits relative to that in the TM-1 fruits at S1, S2, and S3. Nevertheless, one *SPS* (Solyc09g092130.3), four *INVs* (Solyc03g083910.5, Solyc09g010080.3, Solyc09g010090.5.1, and Solyc10g083290.4), two alpha-amylase (*AAMs*; Solyc04g078930.4 and Solyc04g082090.3), one *BAM* (Solyc01g094580.3), one 4-alpha-glucanotransferase (*4-*α*-GTase*; Solyc02g020980.2.1), seven *BGL2s* (Solyc01g081170.3, Solyc04g015560.4, Solyc06g073740.3, Solyc06g073750.4, Solyc06g073760.3, Solyc07g063390.3, Solyc07g063880.4, and Solyc11g071640.3), three *TPSs* (Solyc01g005210.2, Solyc02g071590.3, and Solyc07g006500.3), and two *TPPs* (Solyc06g060600.3 and Solyc08g079060.4) were significantly downregulated in the TM-38 fruits relative to that in the TM-1 fruits at S1, S2, and S3. As for the expression levels of TCA metabolism genes, including two aluminum-activated malate transporter (*ALMT*; Solyc01g096140.3 and Solyc11g068970.2), one isocitrate dehydrogenase (*ICDH*; Solyc01g005560.3), two citrate synthase (*CS*; Solyc07g055840.3 and Solyc12g099260.2), one pyruvate dehydrogenase E1 component subunit beta-1 (*PDHB-1*; Solyc05g024160.3), and two *SDH* (Solyc04g055020.2 and Solyc04g055030.2) were significantly downregulated in the TM-38 fruits relative to that in the TM-1 fruits at the three developmental stages (Figure 7).

### Key Candidate Differentially Expressed Genes Responsible for Organic Acids and Carbohydrate Accumulation in the TM-1 and TM-38 Fruits

To further survey the relationship between genes related to sugar metabolism and the synthesis of organic acids and carbohydrates, correlation analysis was carried out between the transcriptional expression levels of CHO and TCA metabolism genes with the content of organic acids and sugars in the TM-1 and TM-38 fruits at the three developmental stages (Table 2). The results showed that *INV* (Solyc09g010090.5.1 and Solyc10g083290.4), *BGL1* (Solyc10g045240.2), *AAM* (Solyc04g078930.4), *4-*α*-GTase* (Solyc02g020980.2.1), *BGL2* (Solyc01g010390.3, Solyc07g063880.4, and Solyc11g071640.3), *TPS* (Solyc02g071590.3 and Solyc07g006500.3), *TPP* (Solyc06g060600.3, Solyc08g079060.4), *ALMT* (Solyc01g096140.3), and *ACS* (Solyc12g099260.2) were negatively (*r* = −0.819 to −0.986) correlated with D-fructose and D-glucose contents, whereas they positively (*r* = 0.858–0.973) correlated with L-malic acid content. In addition, the content of citric acid was positively (*r* = 0.858–0.973) correlated with *INV* (Solyc09g010080.3 and Solyc09g010090.5.1), *AAM* (Solyc04g082090.3), *4*-α*-GTase* (Solyc02g020980.2.1), *BGL2* (Solyc01g081170.3, Solyc06g073750.4, Solyc06g073760.3, Solyc07g063390.3, and Solyc11g071640.3), *TPS* (Solyc01g005210.2), *TPP* (Solyc08g079060.4), *ALMT* (Solyc01g096140.3 and Solyc11g068970.2), *CS* (Solyc07g055840.3), and *SDH* (Solyc04g055020.2 and Solyc04g055030.2), whereas negatively (*r* = 0.858–0.973) correlated with *SUS* (Solyc07g042550.3), *BAM*, (Solyc08g077530.3), *AGP* (Solyc07g019440.3), and *BGL2* (Solyc09g075060.3). The sugar/organic acid ratio was positively (*r* = 0.885–0.931) correlated with *SUS* (Solyc07g042550.3) and *BAM* (Solyc08g077530.3), while negatively (*r* = −0.823 to −0.918) correlated with *INV* (Solyc09g010080.3 and Solyc09g010090.5.1), *AAM* (Solyc04g082090.3), *4-*α*-GTase* (Solyc02g020980.2.1), *BGL2* (Solyc06g073750.4, Solyc06g073760.3, and Solyc01g081170.3), *TPS* (Solyc01g005210.2 and Solyc07g006500.3), *TPP* (Solyc08g079060.4), and *ALMT* (Solyc01g096140.3).

**FIGURE 8:**
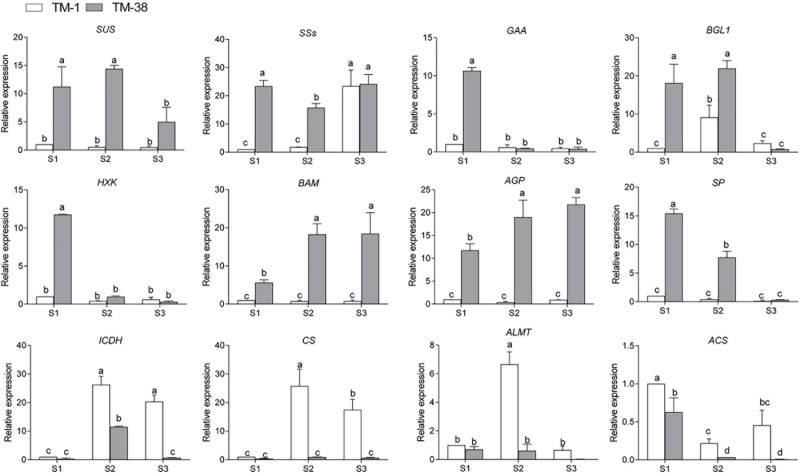
qRT-PCR assay, the mean was calculated from three biological replicates, each with three technical replicates (*n* = 9). These replicates were then normalized relative to the expression of *Actin*.

To further search for candidate genes with major contributions within the complex regulatory networks, the annotation information of all these genes was extracted from the tomato reference genome database. A total of 12 previously used DEGs, including eight genes linked to CHO metabolism, and four genes linked to TCA metabolism shown in Figures 6, 7 were selected for qRT-PCR expression level detection. The general trend of relative expression levels in the three stages was consistent with the in-depth sequencing (Figure 8).

## Discussion

Sugar metabolism plays a key role in plant development, stress response, and yield, which is closely linked to sugar signaling (Ruan, 2014). This coupling is achieved by the production of sugar signaling molecules, such as sucrose, glucose, fructose, and trehalose-6-phosphate, or possibly through signal transduction in the metabolic process itself (O’Hara et al., 2013). Sweetness is particularly appreciated in industrial tomatoes, usually related to fructose and glucose concentrations, mainly accumulated in the vacuoles of the fruit cells (Bona et al., 2016). Although tomato flavor characteristics are the result of complex interactions among multiple metabolites (including more than 400 volatile compounds), the sugar/acid ratio is a key determinant of the sensory experience, which leads to repeated purchase and consumer loyalty (Casals et al., 2011; Beckles, 2012; Hart et al., 2015; Wang et al., 2015). The major organic acids in tomatoes are citric and malic acids, with citric predominating (Marconi et al., 2007). The purpose of this research is to analyze the sugar and organic acid profiles of the TM-1 and TM-38 tomato fruits at three developmental stages, and to characterize genes involved in sugar metabolism.

As the tomato flavor intensity is largely influenced by the interaction of reducing sugars (glucose, fructose) and organic acids (citric and malic acid), which together represent about 60–65% of the dry matter (Bucheli et al., 1999) and the sugar/acid ratio is a key determinant of the sensory experience (Beckles, 2012). In the present research, the fruit Brix of TM-1 (7.6 and 5.7% in 2019 and 2020) was nearly twice that of TM-38 (3.8 and 3.9% in 2019 and 2020) at S3. However, the contents of D-fructose and D-glucose in the TM-38 fruits were significantly higher than those in the TM-1 fruits at S1 and S2, reaching the same level at S3, while the contents of L-malic and citric acid showed an opposite trend at the three developmental stages (Figure 2). The sugar/organic acid ratios of the TM-38 fruits were undoubtedly significantly higher than that of the TM-1 fruits at the three developmental stages. Since TM-38 cultivar is the product of artificial continuous breeding and artificial sweet selection, the sugar/acid ratio of the TM-38 cultivar is higher than that of the wild cultivar TM-1, but it also leads to the loss of flavor substances in TM-1, which eventually leads to a less palatable variety of TM-38. To sum up, these results indicated that the contribution of both L-Malic acid and citric acid to the fruit Brix was much greater than that of glucose and fructose in tomato fruit, or equivalent. Besides, both tomato genotypes had low sucrose content (< 20 μg/g) at all three developmental stages (Data Sheet 1), which may be due to sucrose, which is the photoassimilate transported from the leaves to the fruit of tomato, yet the fruit accumulates predominantly glucose and fructose (Chengappa et al., 1999).

SUS is a highly regulated cytosolic enzyme that catalyzes the reversible conversion of sucrose and UDP into UDP-glucose and fructose (Coleman et al., 2009). Overexpression of *NtSUS3* accelerated the hydrolysis of sucrose and increased fructose content in *Nicotiana tabacum* L. (Daloso et al., 2016). Likewise, two *SUSs* (Solyc03g098290.4 and Solyc07g042550.3) were significantly upregulated in fruits of TM-38 relative to that in TM-1 at the three developmental stages (Figure 6 and Data Sheet 3). Among which, *SUS* (Solyc07g042550.3) was positively (*r* = 0.885) correlated with the sugar/organic acid ratio (Table 2). Thus, we reply that *SUS* (Solyc07g042550.3) contributes to the accumulation of glucose and fructose.

INV is related to the irreversible hydrolysis of sucrose into glucose and fructose. According to the cell location, INV can be classified into cell-wall invertase (CWIN), vacuolar invertase (VIN), and cytoplasmic invertase (CIN) (Wan et al., 2018). A *CWIN* gene named *LIN5* in tomato (*S. lycopersicum* L.) was identified as positively associated with sugar accumulation (Fridman et al., 2004), and in another report, the higher expression of *PlCWIN1* may promote the increase of soluble sugar content (Xue et al., 2019). In sugarcane (*Saccharum* spp. hybrid), leaf chemical formulation *vis-à-vis* reduced soluble acid invertase (SAI) activity and increased sucrose content, which may be due to the significant negative correlation between *SAI* gene and non-reducing sugar (sucrose) in low- and high-sugar genotypes (Jain et al., 2017). Based on the present study, four *INVs* (Solyc03g083910.5, Solyc09g010080.3, Solyc09g010090.5.1, and Solyc10g083290.4), were significantly downregulated in fruits of TM-38 relative to that in TM-1 at the three stages (Figure 6 and Data Sheet 3). Among which, *INVs* (Solyc09g010090.5.1 and Solyc10g083290.4) were negatively (*r* = −0.827 to −0.970) correlated with D-fructose and D-glucose contents, whereas *INV* (Solyc10g083290.4) was positively (*r* = 0.973) correlated with L-malic acid content (Table 2). *INVs* (Solyc09g010080.3 and Solyc09g010090.5.1) were positively (*r* = 0.940) correlated with citric acid content and negatively (*r* = −0.881 to −0.907) correlated with the sugar/organic acid ratio. Therefore, *INV* (Solyc10g083290.4) and *INVs* (Solyc09g010080.3 and Solyc09g010090.5.1) contributed to L-malic acid and citric acid accumulation, respectively. Citric acid may play a more dominant role in the sugar/organic acid ratio.

Plant starch can be synthesized and degraded by several enzyme reactions (Emes et al., 2003; Zeeman et al., 2010). The main source of AGP activity is glucosyl donor ADPG for starch biosynthesis, starch synthase (SS), including SSs and granule-bound SS (GBSS) add glucosyl units at the non-reducing end of linear chains through new α (1→4) linkages (Baroja-Fernandez et al., 2001). BMY is an exoamylase that hydrolyzes a-1,4 glycosidic linkages of polyglucan chains at the non-reducing end to produce maltose during hydrolytic starch degradation (Kaplan et al., 2006; Zanella et al., 2016). Once maltose is exported to the cytosol, it is further metabolized to glucose and/or sucrose and maltodextrins by the activity of cytosolic glucosyltransferases (Tachibana et al., 1997). The high level of gene expression associated with starch degradation (*AAM*, *BAM*, and *SP*) indicated that starch degradation might be a positive process to ensure the sweetness of chestnuts harvest (Zhang et al., 2015). *Arabidopsis* leaves of osmotically stressed *bam1* accumulated more starch and less soluble sugar during the day than wild-type and *bam3* (Zanella et al., 2016). Three *SPs* (Solyc02g077680.4, Solyc03g065340.3, and Solyc05g012510.3), one *SS* (Solyc03g083090.4), two *BAM*s (Solyc08g077530.3 and Solyc09g091030.3), and two *AGP*s (Solyc01g109790.3 and Solyc07g019440.3) were significantly upregulated in fruits of TM-38 relative to that in TM-1 at the three developmental stages. The upregulated expression of these genes was considered to be beneficial to starch degradation and sugar accumulation, while *AAM* (Solyc04g082090.3) was on the contrary, which was negatively (*r* = −0.918) correlated with sugar/organic acid ratio.

Plant glucose can also be synthesized and phosphorylated by several enzyme reactions. For instance, 4-α-GTase (EC 2.4.1.25) could react each maltooligosaccharides (from maltose to maltoheptaose) as the effective substrate to form glucose and various maltooligosaccharides (Tachibana et al., 1997). Likewise, trehalose 6-phosphate is synthesized from UDPG and glucose 6-phosphate *via* TPS, and then dephosphorylated by TPP to yield trehalose, which can be hydrolyzed by trehalase to glucose (Yadav et al., 2014). HXK (EC 2.7.1.1) catalyzes the phosphorylation of hexoses, such as D-glucose, D-fructose, and D-mannose, to hexose 6-phosphate (D-glucose 6-phosphate, D-fructose 6-phosphate, and D-mannose 6-phosphate, respectively) (Menu et al., 2001; Granot, 2007). Three *BGL2s* (Solyc06g005970.2, Solyc06g076780.3, and Solyc09g075060.3), one TPS (Solyc02g072150.3), and three *TPPs* (Solyc03g007290.4, Solyc03g083960.3, and Solyc04g072920.4) were significantly upregulated in fruits of TM-38 relative to that in TM-1 at the three stages and were believed to play a positive role in the synthesis of glucose. Two *HXKs* (Solyc02g091830.3 and Solyc11g065220.2) were also significantly upregulated in fruits of TM-38 relative to that in TM-1 at the three stages and were believed to play an active role in glucose phosphorylation. Besides, *4-*α*-GTase* (Solyc02g020980.2.1), *BGL2* (Solyc06g073750.4, Solyc06g073760.3, and Solyc01g081170.3), *TPS* (Solyc01g005210.2 and Solyc07g006500.3), and *TPP* (Solyc08g079060.4), which were negatively (*r* = −0.823 to −0.918) correlated with the sugar/organic acid ratio, were presumed to have a negative regulatory effect on D-glucose synthesis.

Overall variations in sugar and/or organic acid content are complex metabolic traits that are regulated by gene networks (Famiani et al., 2015). *ALMT*-family genes have been reported to regulate organic acid contents, for example, four *AtALMT9* homologs in grape berries (Terrier et al., 1998), *ALMT II* in apple (Ma et al., 2015), and *ALMT7* in watermelon (Umer et al., 2020). According to these literature data and our findings, the expression level of *ALMT* (Solyc01g096140.3) was positively correlated with citric acid concentration (*r* = 0.943) and the sugar/organic acid ratio (*r* = −0.905) (Table 2), indicating that it is likely to be a key candidate in the gene network of organic acid biosynthesis that contributes to the maximum trait variation.

The characteristics of these genes will improve our understanding of the molecular mechanism of sugar and organic acid biosynthesis. Finally, to evaluate the relative expression level of key putative genes involved in CHO and TCA metabolism, 12 candidate genes including *SUS*, *SSs*, G*AA*, *BGL1*, *HXK*, *BAM*, *AGP*, *SP*, *ICDH*, *CS*, and *ALMT* were selected for qRT-PCR analysis (Figure 8). The results showed the accuracy of transcriptome sequencing. The data collected in this study established a foundation for further investigations to evaluate the structures and functions of the abovementioned genes using molecular biology techniques in the fruit quality of commercially important plants.

## Conclusion

We have investigated the tomato fruit transcriptome of TM-1 (*S. galapagense* L., LA0436) and TM-38 (*S. lycopersicum* L. cultivar M82, LA3475) at three developmental stages and identified specific processes that lead to the variation of fruit Brix. The results indicated that citric acid may play a more dominant role in the sugar/organic acid ratio of tomato fruit, and the contribution of both L-malic acid and citric acid to the fruit Brix was much greater than that of glucose and fructose. The CHO metabolism (ko00500) genes, including *SUS* (Solyc07g042550.3) and *BAM* (Solyc08g077530.3), which were positively (*r* = 0.885–0.931) correlated with the sugar/organic acid ratio, as well as *INV* (Solyc09g010080.3 and Solyc09g010090.5.1), *AAM* (Solyc04g082090.3), *4-*α*-GTase* (Solyc02g020980.2.1), *BGL2* (Solyc06g073750.4, Solyc06g073760.3, and Solyc01g081170.3), *TPS* (Solyc01g005210.2 and Solyc07g006500.3), *TPP* (Solyc08g079060.4), and TCA cycle metabolism (ko00020) gene of *ALMT* (Solyc01g096140.3), which were negatively (*r* = −0.823 to −0.918) correlated with the sugar/organic acid ratio were considered to be the contributing factors of fruit Brix.

## Data Availability Statement

The data presented in the study are deposited in the NCBI Sequence Read Archive (SRA) under the accession number PRJNA744374.

## Author Contributions

HL and YQ designed the research, and reviewed the manuscript. NL and JW collected the experimental data, drafted the manuscript, and carried out the experiments with the help of BW, SH, JH, TY, and PA. All authors read and approved the final manuscript.

## Conflict of Interest

The authors declare that the research was conducted in the absence of any commercial or financial relationships that could be construed as a potential conflict of interest.

## Publisher’s Note

All claims expressed in this article are solely those of the authors and do not necessarily represent those of their affiliated organizations, or those of the publisher, the editors and the reviewers. Any product that may be evaluated in this article, or claim that may be made by its manufacturer, is not guaranteed or endorsed by the publisher.

## Footnotes

1 ftp://ftp.solgenomics.net/tomato_genome/annotation/ITAG4.0_release/)

2
http://www.geneontology.org

3
https://www.genome.jp/kegg/pathway.html

## Supplementary Material

Supplementary Table 1 MS Parameters-UHPLC-HRMS.

Supplementary Table 2 Sequence of primers used for qRT-PCR analysis.

Supplementary Table 3 Statistical table of the number of annotated DEGs in TM-1 vs. TM-38 at three stages.

Supplementary Data Sheet 1 The contents of organic acids and carbohydrates in fruit samples at three stages.

Supplementary Data Sheet 2 The calibration curve of L-Malic acid, Citric acid, D-Fructose, D-Glucose, and D-Sucrose.

Supplementary Data Sheet 3 Gene composition and mean FPKM value of the carbohydrate (ko00500) and citrate cycle (TCA cycle, ko00020) metabolism.

## Abbreviations

*4-* α *-GTase*: 4-alpha-glucanotransferase
*AAM*: alpha-amylase
*ACS*: ATP-citrate synthase alpha chain protein
*AGP*: ADP-glucose pyrophosphorylase large subunit
AGPase: ADP-Glc pyrophosphorylase
*ALMT*: aluminum-activated malate transporter
*BAM*: beta-amylase
*BGL*: beta-glucosidase
CHO: carbohydrate
*CS*: citrate synthase
DEGs: differentially expressed genes
GO: Gene Ontology
HPLC: high-performance liquid chromatography
*HXK*: hexokinase
*ICDH*: isocitrate dehydrogenase
*INV*: invertase
KEGG: Kyoto Encyclopedia of Genes and Genomes
*PDHB-1*: pyruvate dehydrogenase E1 component subunit beta-1
*SDH*: succinate dehydrogenase (ubiquinone) iron–sulfur subunit 3
*SP*: starch phosphorylase
*SPP*: sucrose-phosphatase
*SPS*: sucrose-phosphate synthase
*TPP*: trehalose-phosphate phosphatase
TPS: trehalose-phosphate synthase
TSS: total soluble solids
UDP-glucose: uridine diphosphate glucose
UGPase: uridine diphosphate glucose pyrophosphorylase.

## References

[B1] Barickman T. C. Kopsell D. A. Sams C. E. (2016). Abscisic acid impacts tomato carotenoids, soluble sugars, and organic acids. *HortScience* 51 370–376. 10.21273/hortsci.51.4.370

[B2] Baroja-Fernandez E. Munoz F. J. Akazawa T. Pozueta-Romero J. (2001). Reappraisal of the currently prevailing model of starch biosynthesis in photosynthetic tissues: a proposal involving the cytosolic production of ADP-glucose by sucrose synthase and occurrence of cyclic turnover of starch in the chloroplast. *Plant Cell Physiol.* 42 1311–1320. 10.1093/pcp/pce175 11773523

[B3] Beckles D. M. (2012). Factors affecting the postharvest soluble solids and sugar content of tomato (*Solanum lycopersicum* L.) fruit. *Postharvest. Biol. Tec.* 63 129–140. 10.1016/j.postharvbio.2011.05.016

[B4] Beckles D. M. Hong N. Stamova L. Luengwilai K. (2011). Biochemical factors contributing to tomato fruit sugar content: a review. *Fruits* 67 49–64. 10.1051/fruits/2011066

[B5] Bona E. Cantamessa S. Massa N. Manassero P. Marsano F. Copetta A. (2016). Arbuscular mycorrhizal fungi and plant growth-promoting pseudomonads improve yield, quality and nutritional value of tomato: a field study. *Mycorrhiza* 27 1–11. 10.1007/s00572-016-0727-y 27539491

[B6] Bucheli P. Voirol E. de la Torre R. López J. Rytz A. Tanksley S. D. (1999). Definition of biochemical and molecular markers (quality trait loci) for tomato flavour as tools in breeding. *Acta Hortic.* 487 301–306. 10.17660/actahortic.1999.487.46

[B7] Carlos A. Sabine V. T. Brigitte P. Wilfried R. (2018). Quantification of sugars and organic acids in tomato fruits. *Methods* 5 537–550. 10.1016/j.mex.2018.05.014 30023316 PMC6046607

[B8] Casals J. Pascual L. Cañizares J. Cebolla-Cornejo J. Casañas F. Nuez F. (2011). The risks of success in quality vegetable markets: possible genetic erosion in *Marmande tomatoes* (*Solanum lycopersicum* L.) and consumer dissatisfaction. *Sci. Hortic.* 130 78–84. 10.1016/j.scienta.2011.06.013

[B9] Chengappa S. Guilleroux M. Phillips W. Shields R. (1999). Transgenic tomato plants with decreased sucrose synthase are unaltered in starch and sugar accumulation in the fruit. *Plant Mol. Biol.* 40 213–221. 10412901 10.1023/a:1006136524725

[B10] Çolaka N. G. Ekena N. T. Ülgerb M. Frarya A. Doğanlara S. (2020). Exploring wild alleles from *Solanum pimpinellifolium* with the potential to improve tomato flavor compounds. *Plant Sci.* 298:110567. 10.1016/j.plantsci.2020.110567 32771168

[B11] Coleman H. D. Yan J. Mansfield S. D. (2009). Sucrose synthase affects carbon partitioning to increase cellulose production and altered cell wall ultrastructure. *Proc. Natl. Acad. Sci. U S A.* 106:13118. 10.1073/pnas.0900188106 19625620 PMC2722352

[B12] Daloso D. M. Williams T. C. R. Antunes W. C. Pinheiro D. P. Mueller C. Loureiro M. E. (2016). Guard cell-specific upregulation of sucrose synthase 3 reveals that the role of sucrose in stomatal function is primarily energetic. *New Phytol.* 209 1470–1483. 10.1111/nph.13704 26467445

[B13] Dorais M. Ehret D. L. Papadopoulos A. P. (2008). Tomato (*Solanum lycopersicum*) health components: from the seed to the consumer. *Phytochem. Rev.* 7 231–250. 10.1007/s11101-007-9085-x

[B14] Emes M. J. Bowsher C. G. Hedley C. Burrell M. M. Scrase-Field E. S. Tetlow I. J. (2003). Starch synthesis and carbon partitioning in developing endosperm. *J. Exp. Bot.* 54 569–575. 10.1093/jxb/erg089 12508067

[B15] Famiani F. Battistelli A. Moscatello S. Cruz-Castillo J. G. Walker R. P. (2015). The organic acids that are accumulated in the flesh of fruits: occurrence, metabolism and factors affecting their contents-a review. *Rev. Chapingo Ser. Horticultura* 21 97–128. 10.5154/r.rchsh.2015.01.004

[B16] Finlay M. Dale B. Bradshaw J. E. (2003). Progress in improving processing attributes in potato. *Trends Plant Sci.* 8 310–312. 10.1016/s1360-1385(03)00130-4 12878011

[B17] Fridman E. Carrari F. Liu Y. S. Fernie A. R. Zamir D. (2004). Zooming in on a quantitative trait for tomato yield using interspecific introgressions. *Science* 305 1786–1789. 10.1126/science.1101666 15375271

[B18] Gifford R. M. Thorne J. H. Hitz W. D. Giaquinta R. T. (1984). Crop productivity and photoassimilate partitioning. *Science* 225 801–808. 10.1126/science.225.4664.801 17801136

[B19] Giovannetti M. Avio L. Barale R. Ceccarelli N. Cristofani R. Iezzi A. (2012). Nutraceutical value and safety of tomato fruits produced by mycorrhizal plants. *Br. J. Nutr.* 107 242–251. 10.1017/s000711451100290x 21733294

[B20] Granot D. (2007). Role of tomato hexose kinases. *Funct. Plant Biol.* 34 564–570. 10.1071/fp06207 32689384

[B21] Grof C. P. L. Knight D. P. McNeil S. D. Lunn J. E. Campbell J. A. (1998). A modified assay method shows leaf sucrose-phosphate synthase activity is correlated with leaf sucrose content across a range of sugarcane varieties. *Aust. J. Plant Physiol.* 25 499–502. 10.1071/pp97169

[B22] Hart M. Ehret D. L. Krumbein A. Leung C. Murch S. Turi C. (2015). Inoculation with arbuscular mycorrhizal fungi improves the nutritional value of tomatoes. *Mycorrhiza* 25 359–376. 10.1007/s00572-014-0617-0 25391485

[B23] Hashida Y. Hirose T. Okamura M. Hibara K. Ohsugi R. N. A. (2016). A reduction of sucrose phosphate synthase (SPS) activity affects sucrose/starch ratio in leaves but does not inhibit normal plant growth in rice. *Plant Sci.* 253 40–49. 10.1016/j.plantsci.2016.08.017 27968995

[B24] Jain R. Singh S. P. Singh A. Singh S. Kishor R. Singh R. K. (2017). Soluble acid invertase (SAI) activity and gene expression controlling sugar composition in sugarcane. *Sugar Tech.* 19 669–674. 10.1007/s12355-017-0511-0

[B25] Jawad U. M. Gao L. Gebremeskel H. Safdar L. B. Yuana P. Zhao S. (2020). Expression pattern of sugars and organic acids regulatory genes during watermelon fruit development. *Sci. Hortic.* 265:109102. 10.1016/j.scienta.2019.109102

[B26] Jie Z. Huang C. Yang B. Heikki K. Liu P. Ou S. (2018). Regulation of phytochemicals in fruits and berries by environmental variation-sugars and organic acids. *J. Food Biochem.* 43:e12642. 10.1111/jfbc.12642 31353611

[B27] Kader A. A. (2008). Flavor quality of fruits and vegetables. *J. Sci. Food Agric.* 88 1863–1868.

[B28] Kandel-Kfir M. Damari-Weissler H. German M. A. Gidoni D. Mett A. Belausov E. (2006). Two newly identified membrane-associated and plastidic tomato HXKs: characteristics, predicted structure and intracellular localization. *Planta* 224 1341–1352. 10.1007/s00425-006-0318-9 16761134

[B29] Kaplan F. Sung D. Y. Guy C. L. (2006). Roles of β-amylase and starch breakdown during temperatures stress. *Physiol. Plantarum* 126 120–128. 10.1111/j.1399-3054.2006.00604.x

[B30] Koblitz H. (1991). “Protoplast culture and somatic hybridization in *Lycopersicon*,” in *Genetic Improvement of Tomato*, ed. Kalloo G. (Berlin: Springer).

[B31] Krook J. Vreugdenhil D. Dijkema C. van der Plas L. H. W. (1998). Sucrose and starch metabolism in carrot (*Daucus carota* L.) cell suspensions analysed by 13C-labelling: indications for acytosol and a plastid-localized oxidative pentose phosphate pathway. *J. Exp. Bot.* 49 1917–1924. 10.1093/jxb/49.329.1917 12432039

[B32] Langlois D. Etievant P. X. Pierron P. Jorrot A. (1996). Sensory and instrumental characterization of commercial tomato varieties. *Z Lebensm-Unters Forsch.* 203 534–540. 10.1007/bf01193159

[B33] Lastdrager J. Hanson J. Smeekens S. (2014). Sugar signals and the control of plant growth and development. *J. Exp. Bot.* 65 799–807. 10.1093/jxb/ert474 24453229

[B34] Li D. Mou W. Wang Y. Li L. Mao L. Ying T. (2016). Exogenous sucrose treatment accelerates postharvest tomato fruit ripening through the influence on its metabolism and enhancing ethylene biosynthesis and signaling. *Acta Physiol. Plant* 38:225.

[B35] Livak K. J. Schmittgen T. D. (2001). Analysis of relative gene expression data using real-time quantitative PCR and the 2–ΔΔCT method. *Methods* 25 402–408. 10.1006/meth.2001.1262 11846609

[B36] Ma B. Liao L. Zheng H. Chen J. Wu B. Ogutu C. (2015). Genes encoding aluminum-activated malate transporter II and their association with fruit acidity in apple. *Plant Genome* 8 1–14. 10.3835/plantgenome2015.03.0016 33228269

[B37] Marconi O. Floridi S. Montanari L. (2007). Organic acids profile in tomato juice by HPLC with UV detection. *J. Food Qual.* 30 43–56. 10.1111/j.1745-4557.2007.00105.x

[B38] Menendez C. M. Ritter E. Schafer-Pregl R. Walkemeier B. Kalde A. Salamini F. (2002). Cold sweetening in diploid potato: mapping quantitative trait loci and candidate genes. *Genetics* 162 1423–1434. 10.1093/genetics/162.3.1423 12454085 PMC1462350

[B39] Menu T. Rothan C. Dai N. Petreikov M. Etienne C. Destrac-Irvine A. (2001). Cloning and characterization of a cDNA encoding hexokinase from tomato. *Plant Sci.* 160 209–218. 10.1016/s0168-9452(00)00332-0 11164592

[B40] Ofner I. Lashbrooke J. Pleban T. Aharoni A. Zamir D. (2016). Solanum pennellii backcross inbred lines (BILs) link small genomic bins with tomato traits. *Plant J.* 87 151–160. 10.1111/tpj.13194 27121752

[B41] O’Hara L. E. Paul M. J. Wingler A. (2013). How do sugars regulate plant growth and development? new insight into the role of trehalose-6-phosphate. *Mol. Plant* 6 261–274. 10.1093/mp/sss120 23100484

[B42] Rick C. M. (1974). High soluble-solids content in large-fruited tomato lines derived from a wild green-fruited species. *Hilgardia* 42 493–510. 10.3733/hilg.v42n15p493

[B43] Roby C. Cortes S. Gromova M. LeBail J. L. Roberts J. K. M. (2002). Sucrose cycling in heterotrophic plant cell metabolism: first step towards an experimental model. *Mol. Biol. Rep.* 29 145–149. 12241046 10.1023/a:1020309309045

[B44] Ruan Y. L. (2014). Sucrose metabolism: gateway to diverse carbon use and sugar signaling. *Annu. Rev. Plant Biol.* 65 33–67. 10.1146/annurev-arplant-050213-040251 24579990

[B45] Schaffer A. A. Petreikov M. Miron D. Fogelman M. Spiegelman M. Bnei-Moshe Z. (1999). Modification of carbohydrate content in developing tomato fruit. *HortScience* 34 1024–1027. 10.21273/hortsci.34.6.1024

[B46] Schauer N. Zamir D. Fernie A. R. (2005). Metabolic profiling of leaves and fruit of wild species tomato: a survey of the *Solanum lycopersicum* complex. *J. Exp. Bot.* 56 297–307. 10.1093/jxb/eri057 15596477

[B47] Sweetlove L. Müller-Röber B. Willmitzer L. Hill S. A. (1999). The contribution of adenosine 5-diphosphoglucose pyrophosphorylase to the content of starch synthesis in potato tubers. *Planta* 209 330–337. 10.1007/s004250050640 10502100

[B48] Tachibana Y. Fujiwara S. Takagi M. Imanaka T. (1997). Cloning and expression of the 4-α-glucanotransferase gene from the hyperthermophilic archaeon *Pyrococcus sp*. KOD1, and characterization of the enzyme. *J. Fermentat. Bioeng.* 83 540–548. 10.1016/s0922-338x(97)81134-8

[B49] Terrier N. Deguilloux C. Sauvage F. X. Martinoia E. Romieu C. (1998). Proton pumps and anion transport in *Vitis vinifera*: the inorganic pyrophosphatase plays a predominant role in the energization of the tonoplast. *Plant Physiol. Biochem.* 36 367–377. 10.1016/s0981-9428(98)80078-8

[B50] Umer M. J. Safdar L. B. Gebremeskel H. Zhao S. Yuan P. Zhu H. (2020). Identification of key gene networks controlling organic acid and sugar metabolism during watermelon fruit development by integrating metabolic phenotypes and gene expression profiles. *Hortic. Res.* 7:193. 33328462 10.1038/s41438-020-00416-8 PMC7705761

[B51] Verma S. Sharma V. Kumari N. (2020). Microwave pretreatment of tomato seeds and fruit to enhance plant photosynthesis, nutritive quality and shelf life of fruit. *Postharvest Biol. Tec.* 159:111015. 10.1016/j.postharvbio.2019.111015

[B52] Wan H. Wu L. Yang Y. Zhou G. Ruan Y. L. (2018). Evolution of sucrose metabolism: the dichotomy of invertases and beyond. *Trends Plant Sci.* 23 163–177. 10.1016/j.tplants.2017.11.001 29183781

[B53] Wang L. Baldwin E. A. Zhao W. Plotto A. Sun X. Wang Z. (2015). Suppression of volatile production in tomato fruit exposed to chilling temperature and alleviation of chilling injury by a pre-chilling heat treatment. *LWT-Food Sci. Technol.* 62 115–121. 10.1016/j.lwt.2014.12.062

[B54] Weise S. E. Weber A. P. Sharkey T. D. (2004). Maltose is the major form of carbon exported from the chloroplast at night. *Planta* 218 474–482. 10.1007/s00425-003-1128-y 14566561

[B55] Wind J. Smeekens S. Hanson J. (2010). Sucrose: metabolite and signaling molecule. *Phytochemistry* 71 1610–1614. 10.1016/j.phytochem.2010.07.007 20696445

[B56] Xu S. Sun X. Lu H. Yang H. Ruan Q. Huang H. (2018). Detecting and monitoring the flavor of tomato (*Solanum lycopersicum*) under the impact of postharvest handlings by physicochemical parameters and electronic nose. *Sensors* 18:1847. 10.3390/s18061847 29882769 PMC6021806

[B57] Xue J. Tang Y. Wang S. Xue Y. Liu X. Zhang X. (2019). Evaluation of dry and wet storage on vase quality of cut peony based on the regulation of starch and sucrose metabolism. *Postharvest Biol. Tec.* 155 11–19. 10.1016/j.postharvbio.2019.05.007

[B58] Yadav U. P. Ivakov A. Feil R. Duan G. Y. Walther D. Giavalisco P. (2014). The sucrose–trehalose 6-phosphate (Tre6P) nexus: specificity and mechanisms of sucrose signalling by Tre6P. *J. Exp. Bot.* 65 1051–1068. 10.1093/jxb/ert457 24420566 PMC3935566

[B59] Zanella M. Borghi G. L. Pirone C. Thalmann M. Pazmino D. Costa A. (2016). β-amylase 1 (BAM1) degrades transitory starch to sustain proline biosynthesis during drought stress. *J. Exp. Bot.* 67 1819–1826. 10.1093/jxb/erv572 26792489

[B60] Zeeman S. C. Kossmann J. Smith A. M. (2010). Starch: its metabolism, evolution, and biotechnological modification in plants. *Annu. Rev. Plant Biol.* 61 209–234. 10.1146/annurev-arplant-042809-112301 20192737

[B61] Zhang L. Lin Q. Feng Y. Fan X. Zou F. Yuan D. Y. (2015). Transcriptomic identification and expression of starch and sucrose metabolism genes in the seeds of chinese chestnut (*Castanea mollissima*). *J. Agric. Food Chem.* 63 929–942. 10.1021/jf505247d 25537355

[B62] Zhu Z. Zhang Y. Liu J. Chen Y. Zhang X. (2018). Exploring the effects of selenium treatment on the nutritional quality of tomato fruit. *Food Chem.* 252 9–15. 10.1016/j.foodchem.2018.01.064 29478567

