# Supplementary material for: Identification of the Carbohydrate and Organic Acid Metabolism Genes Responsible for Brix in Tomato Fruit by Transcriptome and Metabolome Analysis
Source: Front Genet. 2021 Sep 3;12:714942. doi: 10.3389/fgene.2021.714942 (PMC8446636; doi:10.3389/fgene.2021.714942)
Supplement: Supplementary Table 1 — MS Parameters-UHPLC-HRMS. [file Table_1.DOC]

**Supplemental Table S1:** MS Parameters-UHPLC-HRMS.

| **Compound Name** | **R.T. (min)** | **m/z** | **Polarity** |
| --- | --- | --- | --- |
| **L-Malic acid** | 0.77 | 133.014 | Negative |
| **Citric acid** | 0.93 | 191.019 | Negative |
| **D-Fructose** | 1.76 | 203.053 | Positive |
| **D-Glucose** | 2.45 | 203.053 | Positive |
| **D-Sucrose** | 3.91 | 365.106 | Positive |
