# Supplementary material for: Identification of the Carbohydrate and Organic Acid Metabolism Genes Responsible for Brix in Tomato Fruit by Transcriptome and Metabolome Analysis
Source: Front Genet. 2021 Sep 3;12:714942. doi: 10.3389/fgene.2021.714942 (PMC8446636; doi:10.3389/fgene.2021.714942)
Supplement: Supplementary Table 2 — Sequence of primers used for qRT-PCR analysis. [file Table_2.DOC]

**Supplemental Table S2: Sequence of primers used for qRT-PCR analysis.**

| **Gene ID** | **Gene name** | **Forward primer (5' to 3')** | **Reverse primer (5' to 3')** |
| --- | --- | --- | --- |
| Solyc07g042550.3 | *SUS* | CTTGGAAGAGCAGGCAGAGA | CAACAGTCAGACCGAAAGCC |
| Solyc01g096140.3 | *ALMT* | GGGAATGCTATGTGGGCTGT | GCAACTCAGTACCTGCCCAT |
| Solyc02g069670.4 | *GAA* | CCCTCTTCTTCTCTTTCCCTCA | CATCGTCATCGTCCACAAATAG |
| Solyc03g119080.4 | *BGL1* | TTGGATTTGCTTATGACCGC | AAGTGTAATGTTGCCCGCA |
| Solyc02g077680.4 | *SP* | CCTCAATCTTCAATCTCAACCG | AAAGGGTCGTGTCTAAAGCGT |
| Solyc01g005560.3 | *ICDH* | ATCTTTGCCTGGACTCGTGG | ATCCAAGAGCCGTTCGTTGT |
| Solyc03g083090.4 | *SSs* | GGTCATCGTGTAATGGTCGTTT | GTTCCAGGTCTGCGGTAAGA |
| Solyc07g055840.3 | *CS* | ATGCACCTGTTTTTCACGCC | TTTGTGCAGCGGTGTTGATG |
| Solyc09g091030.3 | *BAM* | GGAAGGGATTATGATGGATGTG | AACAACCCACCTCGGAAGAG |
| Solyc12g099260.2 | *ACS* | ACACGAGTTTCCGAGCATGT | ATTGTGGTTCAAACGCAGCC |
| Solyc07g019440.3 | *AGP* | TAGGTGGTGGTGTTGGAACTCG | GCAAGGTGACGATTGAGGGA |
| Solyc04g081400.3 | *HK* | TGATAGAGAAATGGATGCCGAG | CCTCAGCACAAATGGAGTGAC |
| Actin |  | GCTGTTCTTTCGCTGTATGC | CATCAAATGGTCAGTCAGGTCA |
