# Supplementary material for: Identification of the Carbohydrate and Organic Acid Metabolism Genes Responsible for Brix in Tomato Fruit by Transcriptome and Metabolome Analysis
Source: Front Genet. 2021 Sep 3;12:714942. doi: 10.3389/fgene.2021.714942 (PMC8446636; doi:10.3389/fgene.2021.714942)
Supplement: Supplementary Table 3 — Statistical table of the number of annotated DEGs in TM-1 vs. TM-38 at three stages. [file Table_3.DOC]

**Supplemental Table S3:** Statistical table of the number of annotated DEGs in TM-1 versus TM-38 at three stages.

| **DEG Set** | **Total** | **COG** | **GO** | **KEGG** | **KOG** | **NR** | **Pfam** | **Swiss-Prot** | **eggNOG** |
| --- | --- | --- | --- | --- | --- | --- | --- | --- | --- |
| TM-1 vs. TM-38 (S1) | 4,819 | 1,924 | 3,445 | 1,672 | 2,361 | 4,774 | 4,016 | 3,730 | 4,252 |
| TM-1 vs. TM-38 (S2) | 4,671 | 1,908 | 3,359 | 1,792 | 2,421 | 4,630 | 3,891 | 3,587 | 4,191 |
| TM-1 vs. TM-38 (S3) | 5,025 | 2,035 | 3,588 | 1,882 | 2,643 | 4,980 | 4,184 | 3,825 | 4,493 |
| TM-1 (S1 vs. S2) | 5,078 | 2,212 | 3,808 | 1,866 | 2,496 | 5,041 | 4,364 | 4,054 | 4,609 |
| TM-1 (S2 vs. S3) | 1,249 | 545 | 928 | 447 | 600 | 1,233 | 1,061 | 1,012 | 1,108 |
| TM-38 (S1 vs. S2) | 3,555 | 1,615 | 2,714 | 1,431 | 1,905 | 3,532 | 3,103 | 2,822 | 3,352 |
| TM-38 (S2 vs. S3) | 763 | 388 | 605 | 334 | 410 | 752 | 669 | 626 | 712 |
